# Supplementary material for: Identification of photoperiod-induced specific miRNAs in the adrenal glands of Sunite sheep (Ovis aries)
Source: Front Vet Sci. 2022 Jul 22;9:888207. doi: 10.3389/fvets.2022.888207 (PMC9354845; doi:10.3389/fvets.2022.888207)
Supplement: Supplementary file 1 [file Data_Sheet_1.ZIP › Supplementary file/miRDeep2 file.zip!/miRDeep2 file/Know_Structure/LP42A3/oar-mir-329a.pdf]

miRBase precursor : oar-mir-329a
Total read count : 163
oar-miR-329a-5p read count : 15
oar-miR-329a-3p read count : 146
remaining reads : 2

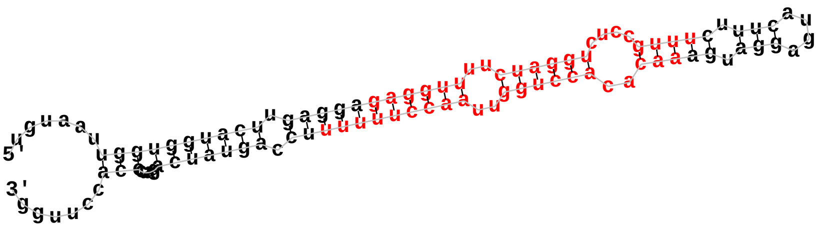

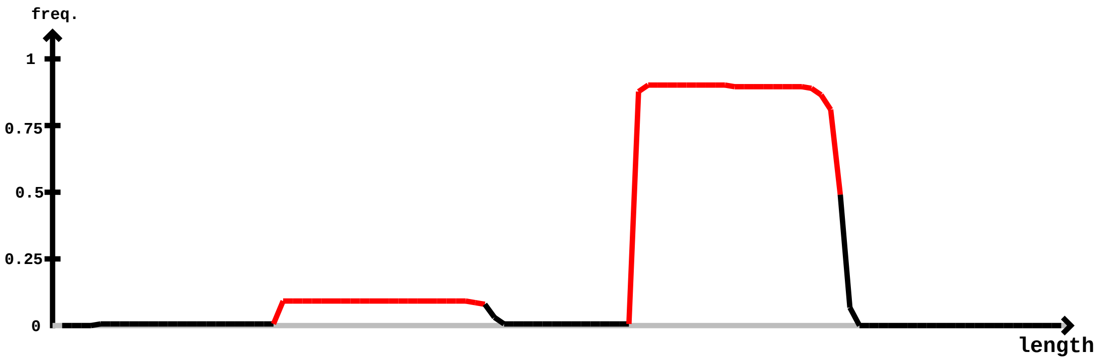


```
                                          oar-miR-329a-5p
                                                                                  oar-miR-329a-3p
5'- uguaauuggugguacuugaggagagguuuucuaggucuccguuucuuucaugaggaugaaacacaccugguuaaccuuuuuuccaguaucagaucccaccuugg   -3'    exp
    .......(((((((((.(((((((((((..((((((....(((((.(((....))).)))))..))))))..)))))))))))).)))).......)))))....    reads     mm     sample
    ...aauuggugguacuugagga..................................................................................    1         0      seq
    ......................gagguuuucuaggucuccgu..............................................................    1         0      seq
    ......................gagguuuucuaggucuccguu.............................................................    1         0      seq
    ......................gagguuuucuaggucuccguuu............................................................    8         0      seq
    ......................gagguuuucuaggucuccguuuc...........................................................    5         0      seq
    .............................................uuucaugaggaugaaacacaccug...................................    1         0      seq
    ...........................................................aacacaccugguuaaccu...........................    1         0      seq
    ...........................................................aacacaccugguuaaccuu..........................    3         0      seq
    ...........................................................Cacacaccugguuaaccuu..........................    1         1      seq
    ...........................................................Cacacaccugguuaaccuuu.........................    1         1      seq
    ...........................................................aacacaccugguuaaccuuu.........................    8         0      seq
    ...........................................................aacacaccugguuaaccuuuu........................    52        0      seq
    ...........................................................aacacaccugguuaaccuuuuu.......................    67        0      seq
    ...........................................................aacacaccugguuaaccuuuuuu......................    9         0      seq
    ............................................................acacaccugguuaaccuuuuu.......................    2         0      seq
    ............................................................acacaccugguuaaccuuuuuu......................    2         0      seq
```
